# Phylogenetic position of the Siberian flying squirrel *Pteromys volans* based on complete mitochondrial genome sequences

**DOI:** 10.1080/23802359.2018.1473728

**Published:** 2018-05-21

**Authors:** Sang Jin Lim, Ki Yoon Kim, Hye Ri Kim, Hee Mun Chae, Yung Chul Park

**Affiliations:** a Division of Forest Science, College of Forest and Environmental Sciences, Kangwon National University, Chuncheon, Republic of Korea;; b Ecosystem Research Division, National Park Research Institute, Korea National Park Service, Wonju, Republic of Korea

**Keywords:** Mitogenome, Siberian flying squirrel, *Pteromys volans*, Sciuridae

## Abstract

The mitogenome of the Siberian flying squirrel *Pteromys volans* is a circular molecule of 16,514 bp, consisting of a control region and a conserved set of 37 genes containing 13 protein-coding genes (PCGs), 22 tRNA genes, and 2 rRNA genes (*12S rRNA* and *16S rRNA*). The mitogenome of the Korean *P. volans* is AT-biased, with a nucleotide composition of 32.2% A, 30.4% T, 12.5% G, and 24.8% C. The phylogenetic analysis revealed that *P. volans* is well placed within the tribe Pteromyini (Sciuridae: Sciurinae), which forms a sister clade to the flying squirrels of the genus *Petaurista*.

The Siberian flying squirrels of *Pteromys volans* (Sciuridae), also known as the Old World flying squirrel, are distributed across a wide range from the Baltic Sea in the west to the Pacific Coast in the east, including the Korean Peninsula and northeast China (Shar et al. 2016). In South Korea, *P. volans* was designated as endangered species as well as natural monument.

We sequenced and characterized the complete mitogenome of a *P. volans* individual caught in Gangneung, South Korea. The voucher specimen (KNPS00027853) was deposited in the National Park Research Institute of Korea National Park Service. Genomic DNA extraction, PCR and gene annotation were conducted according to the previous studies (Jeon and Park 2015; Rahman et al. 2016). A previously published mitogenome of the *P. volans* (JQ230001) was used as reference for gene annotation using Geneious 8.0.5 (Kearse et al. 2012). Phylogenetic tree was constructed under the General Time Reversible model (GTR + R+I) (Nei and Kumar 2000) using maximum-likelihood (ML) procedures implemented in MEGA6 (Tamura et al. 2013). *Glis glis* (NC001892) was used as outgroup.

The complete mitogenome (MH212330) of the *P. volans* contains 16,514 bp in length, which consists of a control region (CR) and a conserved set of 37 vertebrate mitochondrial genes including 13 PCGs, 22 tRNA genes, and 2 rRNA genes (*12S rRNA* and *16S rRNA*). The order and orientation of these genes are identical to those of other mammals (Kim and Park 2016; Kim et al. 2017; Lim et al. 2017).

The mitogenome of the Korean *P. volans* is AT-biased, with a nucleotide composition of 32.2% A, 30.4% T, 12.5% G, and 24.8% C. Total length of the 13 mitochondrial PCGs of the Korean *P. volans* is 11,367 bp long (62.1% A+T), with the exclusion of stop codons, which encode 3789 amino acids. Lengths of two rRNA genes (*12s rRNA* and *16s rRNA*) were 966 bp (61.4% A+T) and 1565 bp long (64.6% A+T), respectively. There are a total of 22 tRNA genes for transferring 20 amino acids, including include two leucine-tRNA genes (*tRNA^Leu(UUR)^* and *tRNA^Leu(CUN)^*) and two serine-tRNA genes (*tRNA^Ser(AGY)^* and *tRNA^Ser(UCN)^*). The mitochondrial *O_R_* is 30 bp long and is located between tRNA^Asn^ and tRNA^Cys^ in the WANCY region. Mitochondrial *CR*, which is located between *tRNA^Pro^* and *tRNA^Phe^*, is 1067 bp long (62.5% A+T).

In intraspecific comparison with the previous published mitogenome of the *P. volans* (JQ230001) which has total size of 16,513 bp, insertion of only a base pair was found in mitochondrial *CR*. Mitogenomes of the two *P. volans* were identical in 99.1% of whole sites of the aligned sequences (16,517 sites). Identical sites of 95.9% were found in mitochondrial *CR* and pairwise identity of 99.3% was found in the other mitochondrial regions except for mitochondrial *CR*.

The phylogenetic analysis revealed that *P. volans* is well placed within the tribe Pteromyini (Sciuridae: Sciurinae), which forms a sister clade to the genus *Petaurista* (Figure 1). The two tribes of Sciurini (*Sciurus vulgaris*) and Marmotini (*Spermophilus dauricus*) formed a sister clade, which is a sister to the tribe Pteromyini.

**Figure 1.**
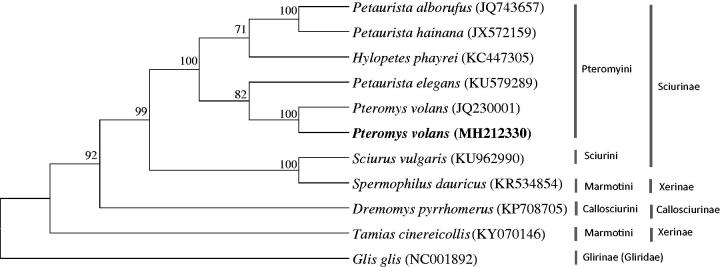
The phylogenetic relationship of *Pteromys volans* and its allied species inferred from maximum-likelihood analysis based on complete mitogenome sequences. The ML tree was generated using the GTR + G+I model, and the robustness of the tree was tested with 1000 bootstrap. The numbers on the branches indicate bootstrap values.
